# SENP1 reduces oxidative stress and apoptosis in renal ischaemia–reperfusion injury by deSUMOylation of HIF‐1α

**DOI:** 10.1111/jcmm.70043

**Published:** 2024-08-28

**Authors:** Yumin Hui, Kang Xia, Jiacheng Zhong, Ye Zhang, Qiangmin Qiu, Zhiyuan Chen, Lei Wang, Xiuheng Liu

**Affiliations:** ^1^ Department of Urology Renmin Hospital of Wuhan University Wuhan China

**Keywords:** apoptosis, HIF‐1α, oxidative stress, renal ischaemia–reperfusion injury, SENP1, SUMOylation

## Abstract

Renal ischaemia–reperfusion injury (RIRI) is a primary cause of acute kidney damage, occurring frequently in situations like renal transplantation, yet the underlying mechanisms were not fully understood. Sentrin‐specific protease 1 (SENP1) is an important member of the SENP family, which is widely involved in various diseases. However, the role of SENP1 in RIRI has been unclear. In our study, we discovered that SENP1 was involved in RIRI and reduced renal cell apoptosis and oxidative stress at elevated levels. Further mechanistic studies showed that hypoxia‐inducible factor‐1α (HIF‐1α) was identified as a substrate of SENP1. Furthermore, SENP1 deSUMOylated HIF‐1α, which reduced the degradation of HIF‐1α, and exerted a renoprotective function. In addition, the protective function was lost after application of the HIF‐1α specific inhibitor KC7F2. Briefly, our results fully demonstrated that SENP1 reduced the degradation of HIF‐1α and attenuated oxidative stress and apoptosis in RIRI by regulating the deSUMOylation of HIF‐1α, suggesting that SENP1 may serve as a potential therapeutic target for the treatment of RIRI.

## 1 INTRODUCTION

Renal ischaemia–reperfusion injury (RIRI) represented a pressing clinical challenge, frequently observed in kidney transplants, large vessel injuries and cases of sepsis.^1^ Initial harm to the kidney arose from reduced or halted renal blood flow during ischaemia, worsened upon reperfusion and potentially progressing into severe renal conditions like acute kidney injury.^2^ Presently, RIRI remained incurable, with oxidative stress and apoptosis identified as key molecular mechanisms.^3^, ^4^ Given the substantial renal insufficiency resulting from this injury and the high mortality rates among patients,^5^, ^6^ there was a growing urgency to comprehensively grasp its pathophysiological processes and continually explore novel therapeutic approaches for its management. Oxidative stress and apoptosis were important pathophysiological processes in the development and pathogenesis of RIRI.^3^, ^4^ When the environment changed such as hypoxia, an imbalance between internal oxidative and antioxidant effects induced oxidative stress. When cells were continuously under pathological stress, apoptotic pathways were triggered, leading to apoptosis.^7^

SUMOylation involved a multi‐step enzymatic cascade reaction that led to subsequent degradation by attaching SUMO proteins to substrates.^8^ This process was reversible, with SENPs capable of removing SUMO couplings from conjugated proteins.^9^ Among these enzymes, SENP1, a crucial member of the SENP family, was widely implicated in various diseases. Research had indicated that inhibiting SENP1 in breast cancer, hepatocellular carcinoma and prostate cancer could impact their proliferation, invasion and migration, and also plays an important role in brain injury, heart injury, lung injury, liver injury.^10^ However, the specific role of SENP1 in RIRI remained unexplored.

HIF‐1α was a vital component of the hypoxia‐inducible factor (HIF‐1) complex, crucial in hypoxia responses and angiogenesis.^11^ Prior research had highlighted its protective role in ischaemia–reperfusion injury (IRI) by activating downstream genes like HO‐1, leading to the production of protective haem.^12^, ^13^, ^14^ The stability of HIF‐1α was intricately regulated through processes such as ubiquitination, phosphorylation, SUMOylation and acetylation.^9^, ^15^ SENP1 had been shown to be critical for the stabilization of HIF‐1α and could increase the stability of HIF‐1α in hypoxia.^16^ SUMOylation played an important role in the regulation of HIF‐1α, but the effect of SUMOylation on HIF‐1α activity had been inconclusive.^16^, ^17^, ^18^ Some studies suggested that HIF‐1α and SUMOylation interact directly with each other,^9^ but another studies had suggested that intermediate molecules mediated their action.^19^ In the context of RIRI, the interplay between SENP1 and HIF‐1α had solely been documented in cisplatin‐induced acute kidney injury (AKI).^20^ Nevertheless, the precise mechanism by which SENP1 modulated HIF‐1α to influence RIRI remained unclear.

In this study, we had clarified the precise mechanism of SENP1 in RIRI. Specifically, SENP1 stabilized HIF‐1α protein through the process of deSUMOylation, leading to antioxidant and anti‐apoptotic effects and attenuating RIRI. SENP1 might be a potential therapeutic strategy for clinical prevention of RIRI.

## 2 MATERIALS AND METHODS

### 2.1 Experimental animals and renal I/R model

Male C57BL/6 mice weighing 20–25 g and averaging 8–9 weeks old were provided by the Experimental Animal Centre of Wuhan University School of Medicine (Wuhan, China) for this study. The mice were kept in a standard laboratory environment with a 12‐h light/12‐h dark cycle, and they had free access to food and water. All experimental protocols were approved by our university's Laboratory Animal Committee and conducted in accordance with the National Institutes of Health Guide for the Care and Use of Laboratory Animals.

The mice were randomly divided into four groups: (1) a sham‐operated group (Sham, *n* = 6); (2) an ischaemia/reperfusion group (I/R, *n* = 6) subjected to 35 min of ischaemia followed by 6 h of reperfusion; (3) another ischaemia/reperfusion group (I/R, *n* = 6) with 35 min of ischaemia and 12 h of reperfusion; (4) and a third ischaemia/reperfusion group (I/R, *n* = 6) exposed to 35 min of ischaemia followed by 24 h of reperfusion. Following isoflurane inhalation anaesthesia, the mice were positioned supine on a thermostatic heating pad set at 37°C. A longitudinal median abdominal incision had made to expose both kidneys and the renal hilum. In the sham‐operated group, the renal hilum was isolated without clamping. Renal ischaemia was induced in the I/R groups by clamping the renal hilum bilaterally using a nontraumatic microvascular clamp for 35 min. After the ischemic period, the clamps were removed to allow for reperfusion for 6/12/24 h, respectively. At the end of the procedure, the mice received subcutaneous rehydration therapy with 20 mL/kg saline, and the median abdominal incision was subsequently closed.

### 2.2 Cell culture and cell hypoxia/reoxygenation (H/R) model

The human renal proximal tubular epithelial cell line (HK2) was procured from the American Type Culture Collection (ATCC, USA) and cultivated in DMEM/F12 medium (Sigma‐Aldrich). The medium was supplemented with 10% fetal bovine serum (Gibco) and 1% penicillin–streptomycin, and the cells were maintained in a 37°C incubator with 5% CO_2_. To induce hypoxic injury, HK2 cells were subjected to hypoxia for 12 h (1% O_2_, 94% N_2_ and 5% CO_2_) in a glucose‐free, serum‐free medium. Following this, complete medium was reintroduced, and the cells were transferred to a normoxic incubator (5% CO_2_ and 95% air) for 2 h, 4 h and 6 h. Control cells were incubated in standard culture medium within a regular incubator (5% CO_2_ and 95% air).

### 2.3 Measurement of kidney function

Blood was collected from the heart and centrifuged at 3000 rpm for 10 min at 4°C. The resulting supernatant was used for biochemical analysis. Blood creatinine (Cr) and urea nitrogen (BUN) levels were determined spectrophotometrically following the instructions provided in a commercial kit (BioSharp, Hefei, China).

### 2.4 Histological staining

Mice kidneys, fixed in paraformaldehyde and embedded in paraffin, were sliced into 4‐μm sections. These sections were then deparaffinized, stained with haematoxylin and eosin, and meticulously examined under a microscope to assess their histological features.

Renal histological evaluation was conducted blindly by two independent experienced pathologists using a standardized grading scale ranging from 0 to 4: (0) indicating no damage, (1) denoting ≤25% tissue involvement, (2) indicating between 25% and 50%, (3) representing 50%–75%, and (4) signifying ≥75%. Renal impairment was characterized by features such as tubular dilatation, necrosis, loss of brush border, detachment of tubular epithelial cells and tubular formation.^21^

### 2.5 Immunohistochemistry

Mice kidneys embedded in paraffin were sliced into 4 μm sections, deparaffinized and blocked for nonspecific binding. Specific antibodies, anti‐SENP1 (Proteintech, 25349‐1‐AP, 1:200) and anti‐Caspase3/C‐Caspase3 (Proteintech, 66470‐2‐Ig, 1:600), were applied to the kidney tissue sections. After incubation with secondary antibodies, the sections were treated with DAB solution and examined under an inverted microscope. Image analysis was conducted using Image‐Pro Plus (version 6.0).

### 2.6 Immunofluorescence

Paraffin sections were deparaffinized and treated with 0.5% Triton X‐100 for 15 min. Subsequently, the sections were sealed with blocking serum for 1 h at room temperature. After removing the blocking solution, primary antibodies of appropriate concentrations anti‐SUMO1 (Proteintech, 10329‐1‐AP, 1:100) and anti‐HIF‐1α (Proteintech, 20960‐1‐AP, 1:200) were applied and left overnight in a wet box at 4°C. The sections were then washed with PBS, followed by the addition of fluorescent secondary antibodies in a wet box at room temperature for 1 h, shielded from light. DAPI staining was performed for 15 min in a darkened wet box. To prevent fluorescence quenching, sections were sealed with adhesive containing an antifluorescent quencher. Between each reagent change, sections were washed with PBS and stored at 37°C. Microscopic observations were made after washing the samples with PBS, drying and storing them at 37°C. Fluorescence analysis was conducted using ImageJ software (NIH, Bethesda, MD, USA).

### 2.7 TdT‐mediated dUTP nick‐end labelling (TUNEL)

Apoptosis in renal tissue was assessed following the manufacturer's instructions for the TUNEL Assay Kit (C1090, Beyotime Biotechnology). Briefly, paraffin sections were completely dewaxed, treated with proteinase K and then incubated with TUNEL working solution at 37°C in a light‐protected incubator for 60 min. Subsequently, DAPI working solution was applied, and the sections were dehydrated and sealed with an anti‐fluorescence quenching sealer for observation.

### 2.8 Reactive oxygen species (ROS) measurement

In the in vivo assessments, ROS levels were gauged using the dihydroethidium (DHE) kit (Beyotime Biotechnology, #S0063). Tissues pre‐treated with the compounds were loaded with the respective probe. Rosup was introduced as a positive control, and fluorescence microscopy was employed for analysis.

### 2.9 Quantitative real‐time PCR

RNA extraction was carried out from HK2 cells or frozen kidney tissues using RNAiso Plus (TaKaRa Biotech, Dalian, China). Subsequently, RNA was reverse transcribed into cDNA utilizing the PrimeScript™ RT Reagent Kit with gDNA Eraser (Takara). Quantitative real‐time PCR analysis was conducted employing an ABIViiA7DX System (Foster City, CA, USA) with GAPDH serving as the internal control. The relative gene expression levels were determined using the 2‐ΔΔCT method. For primer sequences, refer to Table S1.

### 2.10 Western blot analysis

Kidney tissues or HK2 cells were lysed using RIPA lysis buffer (Beyotime Biotechnology) supplemented with a protease inhibitor cocktail. After centrifugation, the lysates were collected. Protein concentrations were determined, and samples were separated on SDS–polyacrylamide gels and transferred to nitrocellulose (NC) membranes. Membranes were incubated with primary antibodies overnight at 4°C. The primary antibodies included anti‐SENP1 (Proteintech, 25349‐1‐AP, 1:1000), anti‐HIF‐1α (Proteintech, 20960‐1‐AP, 1:2000), anti‐Bax (Proteintech, 50,599‐2‐Ig, 1:2000), anti‐Bcl2 (Proteintech, 26593‐1‐AP, 1:1000), anti‐Caspase3/C‐Caspase3 (Proteintech, 66470‐2‐Ig, 1:1000), anti‐SOD1 (Proteintech, 10269‐1‐AP, 1:5000), anti‐SOD2 (Proteintech, 24127‐1‐AP, 1:5000), anti‐Catalase (Proteintech, 21260‐1‐AP, 1:2000), anti‐GAPDH (Proteintech, 10494‐1‐AP, 1:5000), anti‐SUMO1 (Proteintech, 10329‐1‐AP, 1:5000), anti‐Flag (Servicebio, GB11938, 1:1000) and anti‐HA (Servicebio, GB12939, 1:1000). Subsequently, the appropriate secondary antibodies were applied. The bands were visualized using a protein blotting detection system and quantified using ImageJ software (NIH, Bethesda, MD, USA).

### 2.11 Flow cytometry

To assess the impact of various treatments on apoptosis and oxidative stress, cells were stained using Annexin V‐FITC/PI and DCFH‐DA detection kits as per the manufacturer's guidelines, and subsequently analysed through flow cytometry. Flow cytometry data were processed using BD FACSDiva software v7.0 from Becton‐Dickinson, USA.

### 2.12 Transfection

The recombined adenoviruses mediating SENP1 overexpression and knockdown were constructed as previously described.^22^ The recombined adenovirus vector was applied as negative control. To inhibit HIF‐1α activity in vivo, KC7F2 (Selleck) were injected intraperitoneally at a concentration of 50 mg/kg for 3 consecutive days before surgery in mice. DMSO was applied as negative control.

The lentiviruses mediating SENP1 overexpression and knockdown were generated as outlined in a previous study.^23^ For in vitro inhibition of HIF‐1α activity, KC7F2 (Selleck) was administered at a concentration of 5 μM for 24 h before H/R treatment. DMSO served as the negative control.

### 2.13 Immunoprecipitation (IP)

Protein samples from specific experiments were incubated with relevant antibodies overnight. Subsequently, Protein A/G agarose beads were introduced and agitated for 2 h at 4°C. The proteins bound to the beads were then eluted and analysed via immunoblotting using the designated antibodies. The antibodies used were consistent with the Western blot analysis.

### 2.14 Statistical analysis

Statistical analyses were conducted using GraphPad Prism software (version 8.0, USA) through unpaired *t*‐tests or one‐way ANOVA, as appropriate. All values are expressed as mean ± standard deviation (SD). Statistical significance was defined as *p* < 0.05.

## 3 RESULTS

### 3.1 The expression of SENP1 increases during RIRI

**FIGURE 1:**
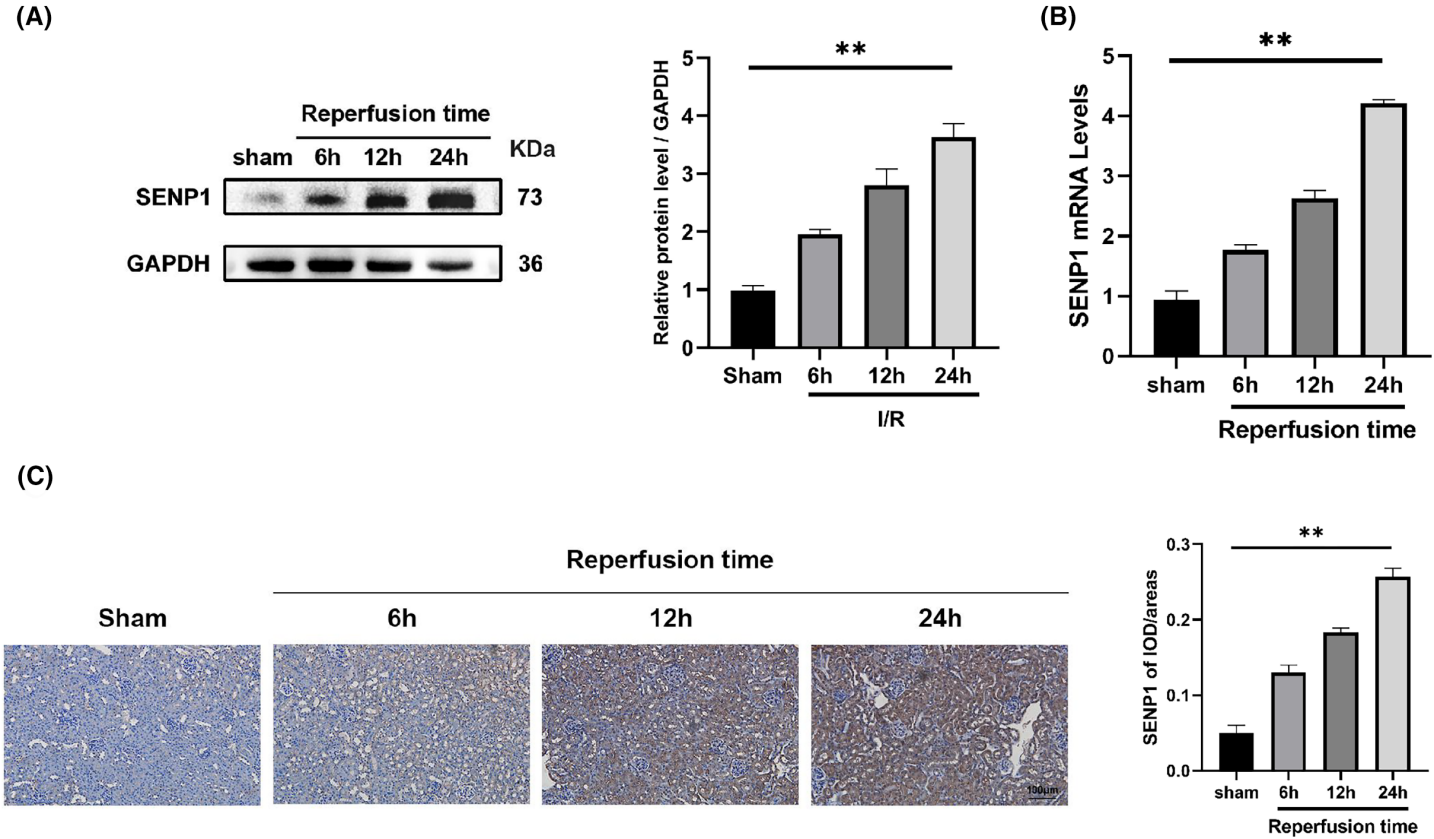
The expression of SENP1 increases during RIRI. (A) WB detection of SENP1 protein levels. (B) qPCR detection of SENP1 mRNA levels. (C) Representative images immunohistochemistry of SENP1 in mice nephridial tissues (left) and related quantitative analysis (right). Bar = 100 μm. Values are expressed as the mean ± SEM. *N* = 3–5. **p < 0.01.

Initially, we investigated the expression of SENP1 in renal tissues. As shown in Figure 1A,B, both the mRNA and protein levels of SENP1 exhibited an increase in the RIRI group compared to the Sham group. Furthermore, the expression of SENP1 showed a significant elevation with the extension of reperfusion time (6 h vs. 12 h vs. 24 h). Immunohistochemical results revealed conspicuous positive staining for SENP1 in the RIRI group as opposed to the sham group, with density intensifying as reperfusion time increased (Figure 1C). These outcomes strongly suggested the potential involvement of SENP1 in the progression of RIRI. Accordingly, we chose the reperfusion time (24 h) to carry out all following experiments.

### 3.2 Knockdown of SENP1 exacerbated apoptosis and oxidative stress induced by RIRI

**FIGURE 2:**
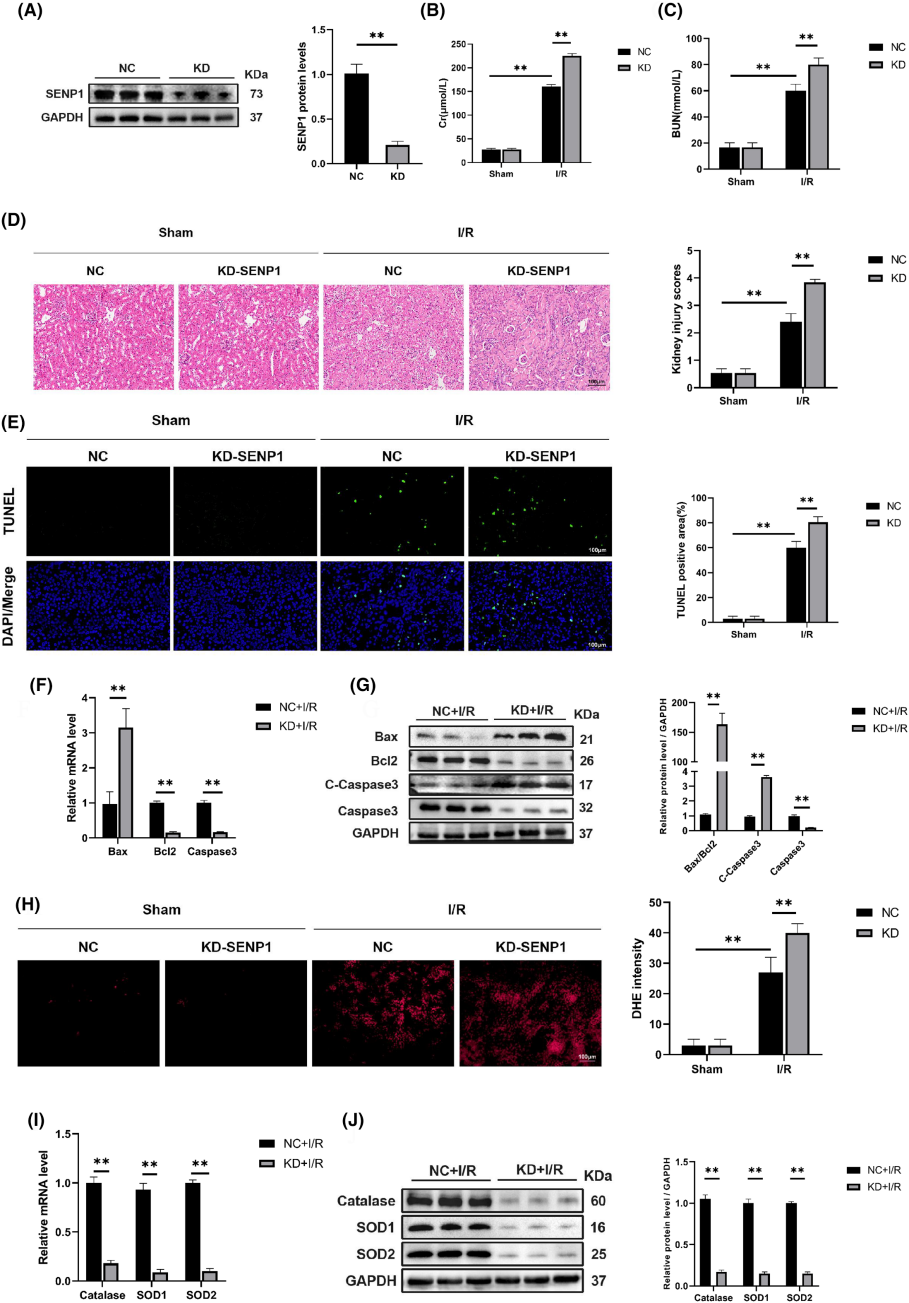
Knockdown of SENP1 exacerbated apoptosis and oxidative stress induced by RIRI (24 h of reperfusion). (A) WB detection of SENP1 protein levels. (B, C) Detection of Cr and BUN in mouse serum. (D, E) Representative images of haematoxylin and eosin staining and representative images of TUNEL staining in mice nephridial tissues and related quantitative analysis. Bar = 100 μm. (F) qPCR detection of Bax, Bcl2 and Caspase3 mRNA levels. (G) WB detection of Bax, Bcl2, C‐Caspase3 and Caspase3 protein levels. (H) Representative images of DHE staining in mice nephridial tissues and related quantitative analysis. (I) qPCR detection of Catalase, SOD1 and SOD2 mRNA levels. (J) WB detection of Catalase, SOD1 and SOD2 protein levels. Values are expressed as the mean ± SEM. *N* = 3. **p < 0.01.

To assess the impact of SENP1 downregulation on RIRI, we developed a mouse model with SENP1 knockdown. As depicted in Figure 2A, during renal ischaemia, the knockdown model significantly reduced SENP1 expression. Subsequently, serum analysis revealed that SENP1 knockdown exacerbated renal injury induced by ischaemia–reperfusion (Figure 2B,C). Histological examination using HE staining indicated more pronounced renal tissue damage and inflammatory cell infiltration in the renal tissues of SENP‐knockdown mice during RIRI (Figure 2D). TUNEL staining demonstrated an increased rate of apoptosis with SENP1 knockdown (Figure 2E). Additionally, examination of relevant proteins and mRNAs revealed that SENP1 knockdown heightened the expression of apoptotic molecules (Figure 2F,G). Further analysis through DHE staining and quantitative protein assessment indicated that SENP1 knockdown elevated oxidative stress levels during RIRI (Figure 2H–J). These findings collectively suggested a crucial role for SENP1 in mitigating RIRI.

### 3.3 Overexpression of SENP1 attenuated apoptosis and oxidative stress induced by RIRI

**FIGURE 3:**
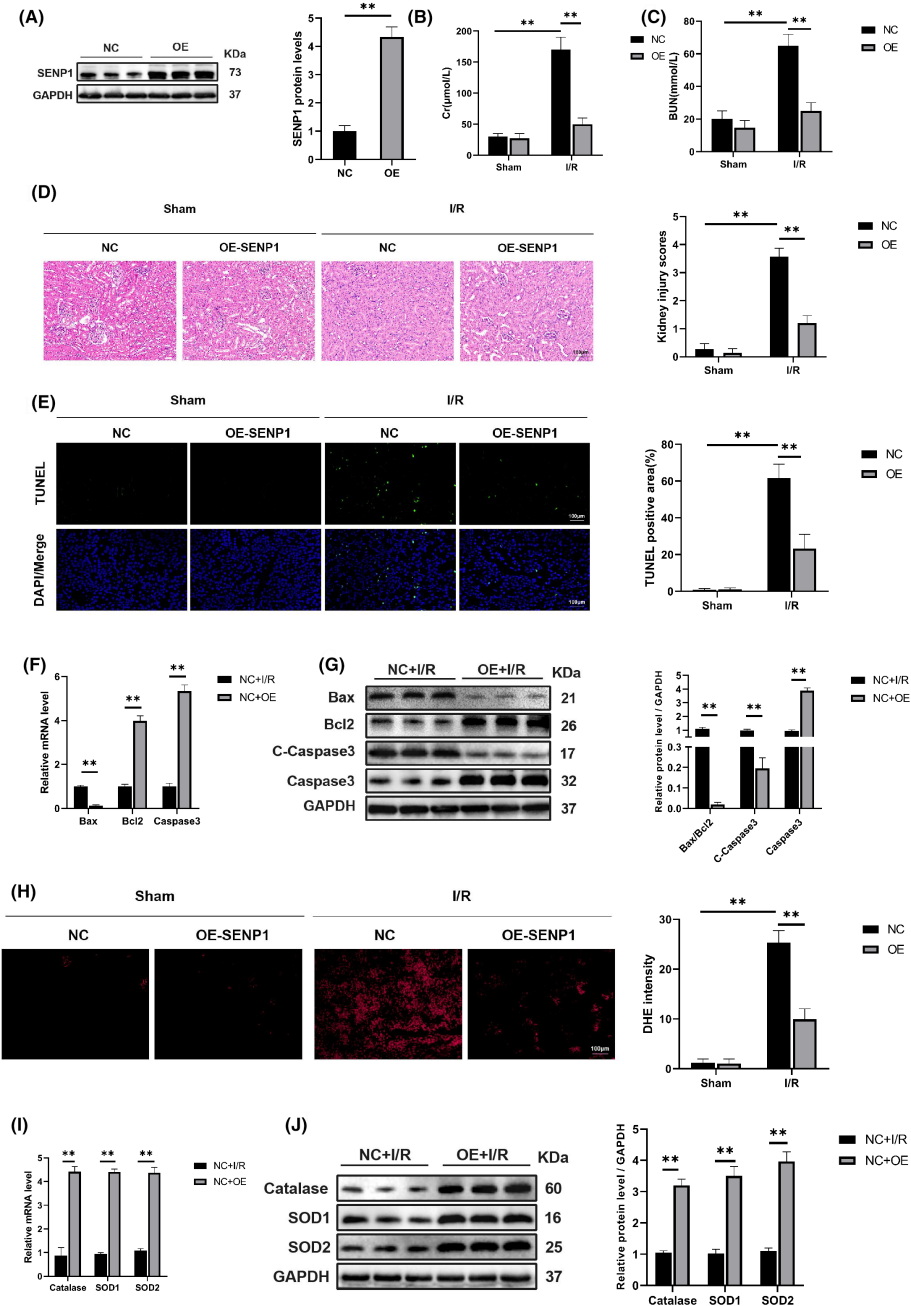
Overexpression of SENP1 exacerbated apoptosis and oxidative stress induced by RIRI (24 h of reperfusion). (A) WB detection of SENP1 protein levels. (B, C) Detection of Cr and BUN in mouse serum. (D, E) Representative images of haematoxylin and eosin staining and representative images of TUNEL staining in mice nephridial tissues and related quantitative analysis. Bar = 100 μm. (F) qPCR detection of Bax, Bcl2 and Caspase3 mRNA levels. (G) WB detection of Bax, Bcl2, C‐Caspase3 and Caspase3 protein levels. (H) Representative images of DHE staining in mice nephridial tissues and related quantitative analysis. (I) qPCR detection of Catalase, SOD1 and SOD2 mRNA levels. (J) WB detection of Catalase, SOD1 and SOD2 protein levels. Values are expressed as the mean ± SEM. *N* = 3. **p < 0.01.

To investigate the impact of SENP1 overexpression on RIRI, we generated a mouse model with heightened SENP1 expression. As depicted in Figure 3A, the overexpression model significantly elevated SENP1 levels during RIRI. Subsequently, we observed a marked reduction in serum Cr and BUN levels, as illustrated in Figure 3B,C. Histological examination using HE staining revealed that SENP1 overexpression effectively mitigated renal tissue injury in mice subjected to RIRI (Figure 3D). TUNEL and DHE staining further demonstrated that SENP1 overexpression led to a decrease in apoptosis rates and oxidative stress levels, as depicted in Figure 3E,H. Consistent with these findings, both protein and mRNA analyses (Figure 3F,G,I,J) affirmed the protective role of SENP1 in ameliorating RIRI. These collective results underscored the significant contribution of SENP1 in alleviating the detrimental effects of RIRI.

### 3.4 SENP1 regulated H/R‐induced apoptosis and oxidative stress in HK2 cells

**FIGURE 4:**
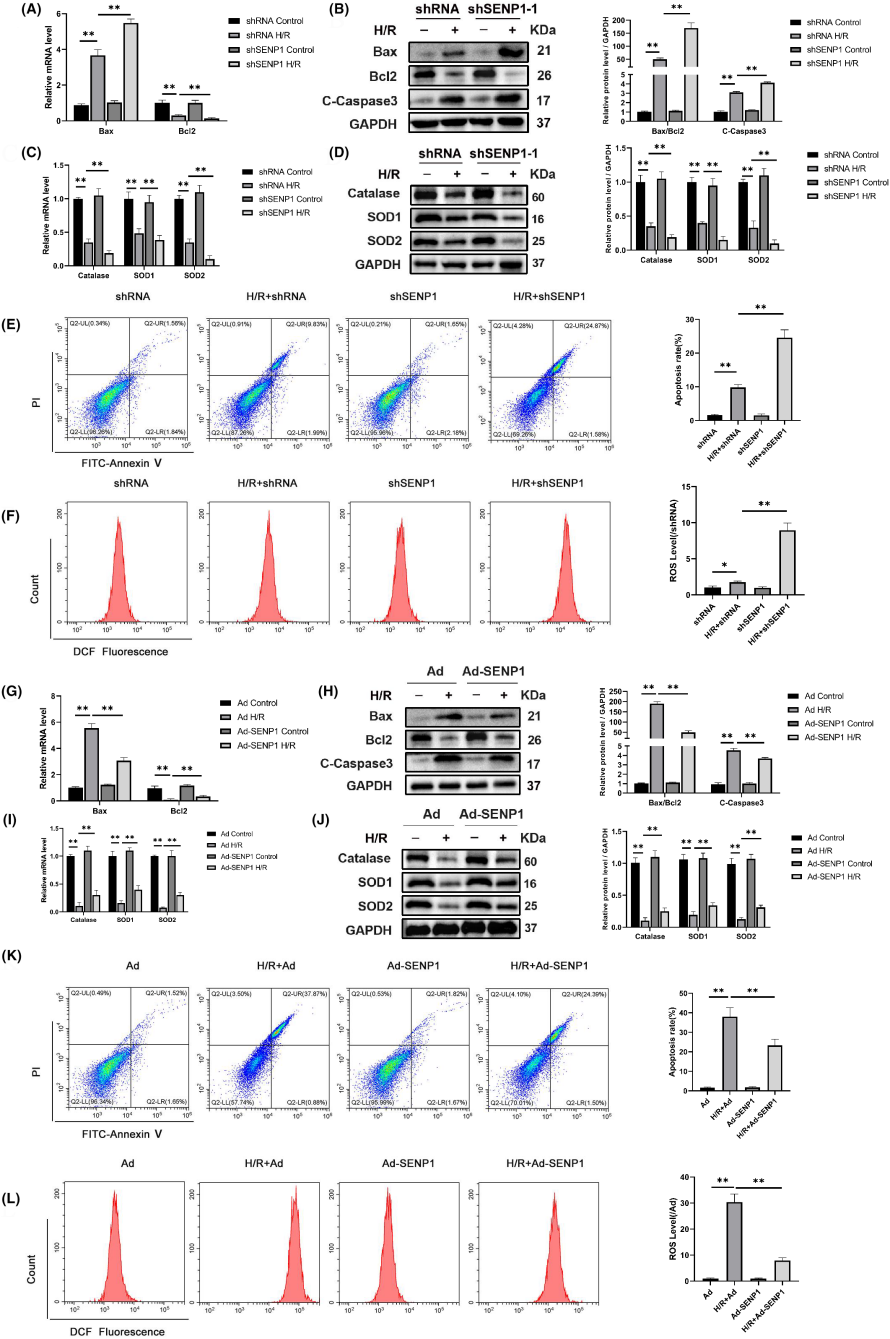
SENP1 regulated H/R‐induced apoptosis and oxidative stress in HK2 cells. (A) qPCR detection of Bax and Bcl2 mRNA levels. (B) WB detection of Bax, Bcl2 and C‐Caspase3 protein levels. (C) qPCR detection of catalase, SOD1 and SOD2 mRNA levels. (D) WB detection of Catalase, SOD1 and SOD2 protein levels. (E) Representative images of flow cytometry to detect the apoptosis rate of HK2 cells (left) and related quantitative analysis (right). (F) Representative images of flow cytometry to detect the ROS level of HK2 cells (left) and related quantitative analysis (right). (G) qPCR detection of Bax and Bcl2 mRNA levels. (H) WB detection of Bax, Bcl2 and C‐Caspase3 protein levels. (I) qPCR detection of Catalase, SOD1 and SOD2 mRNA levels. (J) WB detection of Catalase, SOD1 and SOD2 protein levels. (K) Representative images of flow cytometry to detect the apoptosis rate of HK2 cells (left) and related quantitative analysis (right). (L) Representative images of flow cytometry to detect the ROS level of HK2 cells (left) and related quantitative analysis (right). Values are expressed as the mean ± SEM. *N* = 3. **p < 0.01. *p < 0.05.

We proceeded to establish an in vitro model to validate our hypothesis. Notably, both apoptosis and ROS levels in HK2 cells exhibited a significant increase during H/R.^7^ Knocking down SENP1 resulted in elevated ROS and apoptosis levels in HK2 cells compared to the H/R group (refer to Figure 4E,F). Additionally, a further decrease in the levels of associated antioxidant enzymes was observed, accompanied by corresponding alterations in apoptotic molecule levels (refer to Figure 4A–D). Conversely, HK2 cells overexpressing SENP1 demonstrated heightened resistance to ROS and apoptosis during H/R (refer to Figure 4K,L). The decline in antioxidant enzyme expression induced by H/R was reversed through SENP1 overexpression, correlating with changes in apoptotic molecule levels (refer to Figure 4G–J). These findings underscored the role of SENP1 in the in vitro H/R process.

### 3.5 HIF‐1α mediated the protective effect of SENP1 against RIRI

**FIGURE 5:**
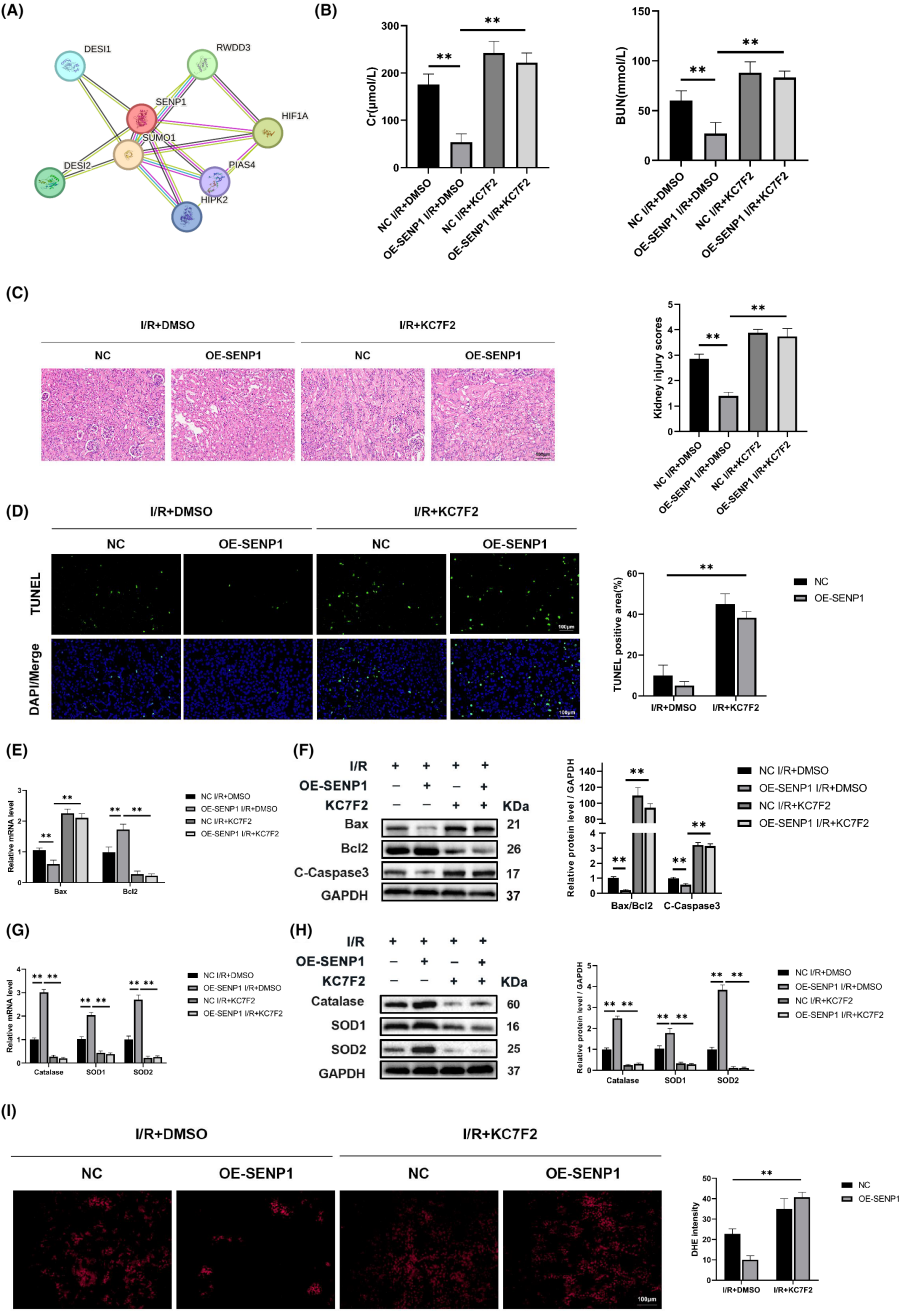
HIF‐1α mediates the Protective Effect of SENP1 against RIRI (24 h of reperfusion). (A) Analysis of the interaction between SENP1 and HIF‐1α protein. (B) Detection of Cr and BUN in mouse serum. (C, D)Representative images of haematoxylin and eosin staining and representative images of TUNEL staining in mice nephridial tissues and related quantitative analysis. Bar = 100 μm. (E) qPCR detection of Bax and Bcl2 mRNA levels. (F) WB detection of Bax, Bcl2 and C‐Caspase3 protein levels. (G) qPCR detection of Catalase, SOD1 and SOD2 mRNA levels. (H) WB detection of Catalase, SOD1 and SOD2 protein levels. (I) Representative images of DHE staining in mice nephridial tissues and related quantitative analysis. Values are expressed as the mean ± SEM. *N* = 3. **p < 0.01.

In order to identify the substrates of SENP1 in renal ischaemia–reperfusion injury, we screened the interacting molecules of SENP1 in the STRING database (Figure 5A), among which HIF‐1α, as an extremely important regulator in renal ischaemia–reperfusion injury, was identified as the target for the next study. To examine the influence of HIF‐1α on SENP1‐mediated protection against RIRI, we employed a specific HIF‐1α inhibitor (KC7F2) for further investigations. The results revealed that the overexpression of SENP1 provided a protective effect against RIRI, as depicted in Figure 5B,C. However, this protective effect was reversed by KC7F2. The outcomes of the TUNEL staining assay and protein blotting indicated that the anti‐apoptotic effect induced by SENP1 overexpression was similarly inhibited (Figure 5D–F). Additionally, KC7F2 significantly reduced the anti‐oxidative stress effect conferred by SENP1 overexpression (Figure 5G–I). These findings collectively suggested that the anti‐apoptotic and anti‐oxidative stress capabilities of SENP1 were mediated by HIF‐1α.

### 3.6 HIF‐1α mediated the protection of SENP1 against apoptosis and oxidative stress in the H/R

**FIGURE 6:**
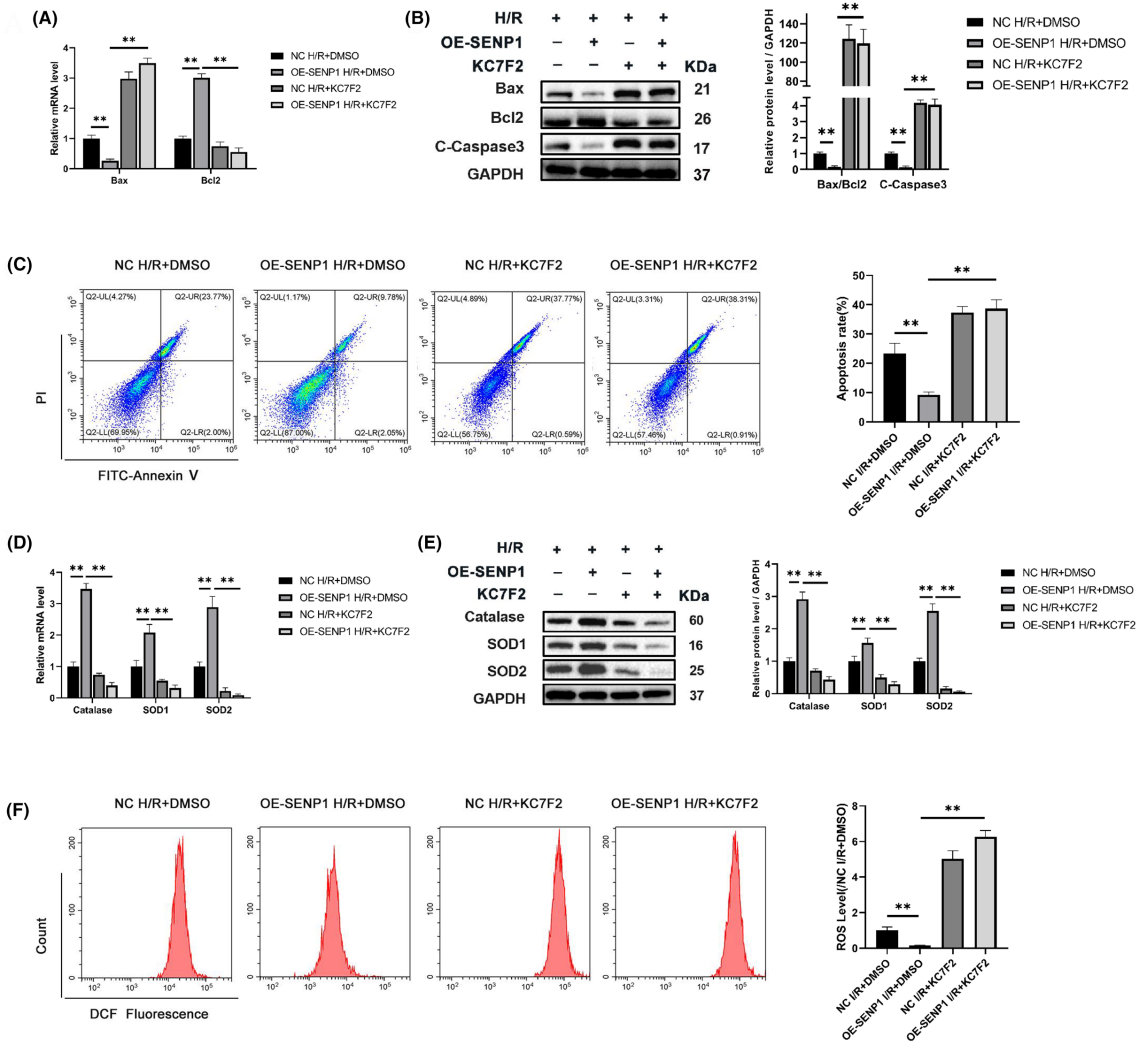
HIF‐1α mediates the protection of SENP1 against apoptosis and oxidative stress in the H/R. (A) qPCR detection of Bax and Bcl2 mRNA levels. (B) WB detection of Bax, Bcl2 and C‐Caspase3 protein levels. (C) Representative images of flow cytometry to detect the apoptosis rate of HK2 cells (left) and related quantitative analysis (right). (D) qPCR detection of Catalase, SOD1 and SOD2 mRNA levels. (E) WB detection of Catalase, SOD1 and SOD2 protein levels. (F) Representative images of flow cytometry to detect the ROS level of HK2 cells (left) and related quantitative analysis (right). Values are expressed as the mean ± SEM. *N* = 3. **p < 0.01.

In vitro, the overexpression of SENP1 similarly demonstrated protective effects against H/R. Protein blotting results revealed that KC7F2 reversed the anti‐apoptotic effect induced by SENP1 overexpression (Figure 6A,B), as further supported by flow cytometry results (Figure 6C). Additionally, KC7F2 also impeded the anti‐oxidative stress effect of SENP1, as demonstrated in Figure 6D–F. These outcomes consistently aligned with the in vivo experiments, collectively indicating that HIF‐1α played a pivotal role in SENP1‐mediated regulation of RIRI.

### 3.7 SENP1 targeted the deSUMOylation of HIF‐1α

**FIGURE 7:**
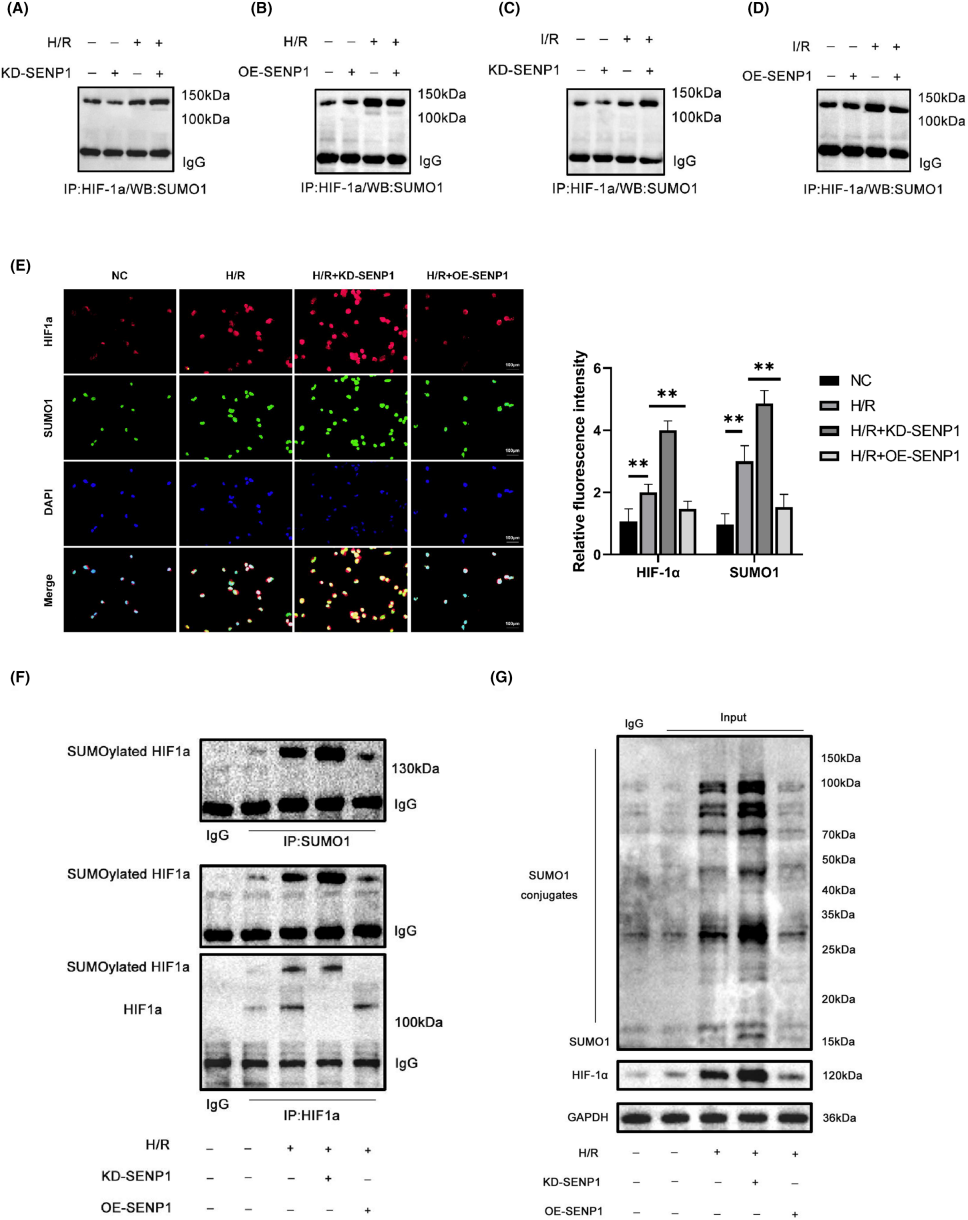
SENP1 targets the deSUMOylation of HIF‐1α. (A, B) WB detection of HIF‐1α and SUMOylation protein levels in vitro. (C, D) WB detection of HIF‐1α and SUMOylation protein levels in vivo (24 h of reperfusion). (E) Representative images of immunofluorescence to detect HIF‐1α (red), SUMO1 (green), and DAPI (blue) in HK2 cells. Bar = 20 μm. (F, G) IP detection of the interaction between HIF‐1α and SUMO1. Values are expressed as the mean ± SEM. *N* = 3. **p < 0.01.

HIF‐1α played a crucial role in RIRI regulation.^24^ Existing evidence indicated that SENP1 had the potential to modulate HIF‐1α.^9^ We posited that SENP1 influenced RIRI by regulating HIF‐1α. To test this hypothesis, we assessed the expression of HIF‐1α and SUMOylation during H/R. Our results revealed that H/R led to an augmentation in SUMOylation and an increase in HIF‐1α expression. Notably, the knockdown of SENP1 significantly intensified these changes during H/R, as observed in Figure 7A,B. Consistently, in vivo experiments using the RIRI model yielded similar outcomes (Figure 7C,D). Immunofluorescence analysis demonstrated a notable reduction in the binding of HIF‐1α to SUMO1 in the SENP1 overexpression group compared to the H/R group. Conversely, this binding increased with SENP1 knockdown (Figure 7E). Subsequent immunoprecipitation (IP) experiments corroborated that SENP1 regulated the interaction between SUMO1 and HIF‐1α during H/R. In summary, SENP1 overexpression diminished H/R‐induced SUMOylation of HIF‐1α, while SENP1 knockdown yielded the opposite effect (Figure 7F,G).

## 4 DISCUSSION

**FIGURE 8:**
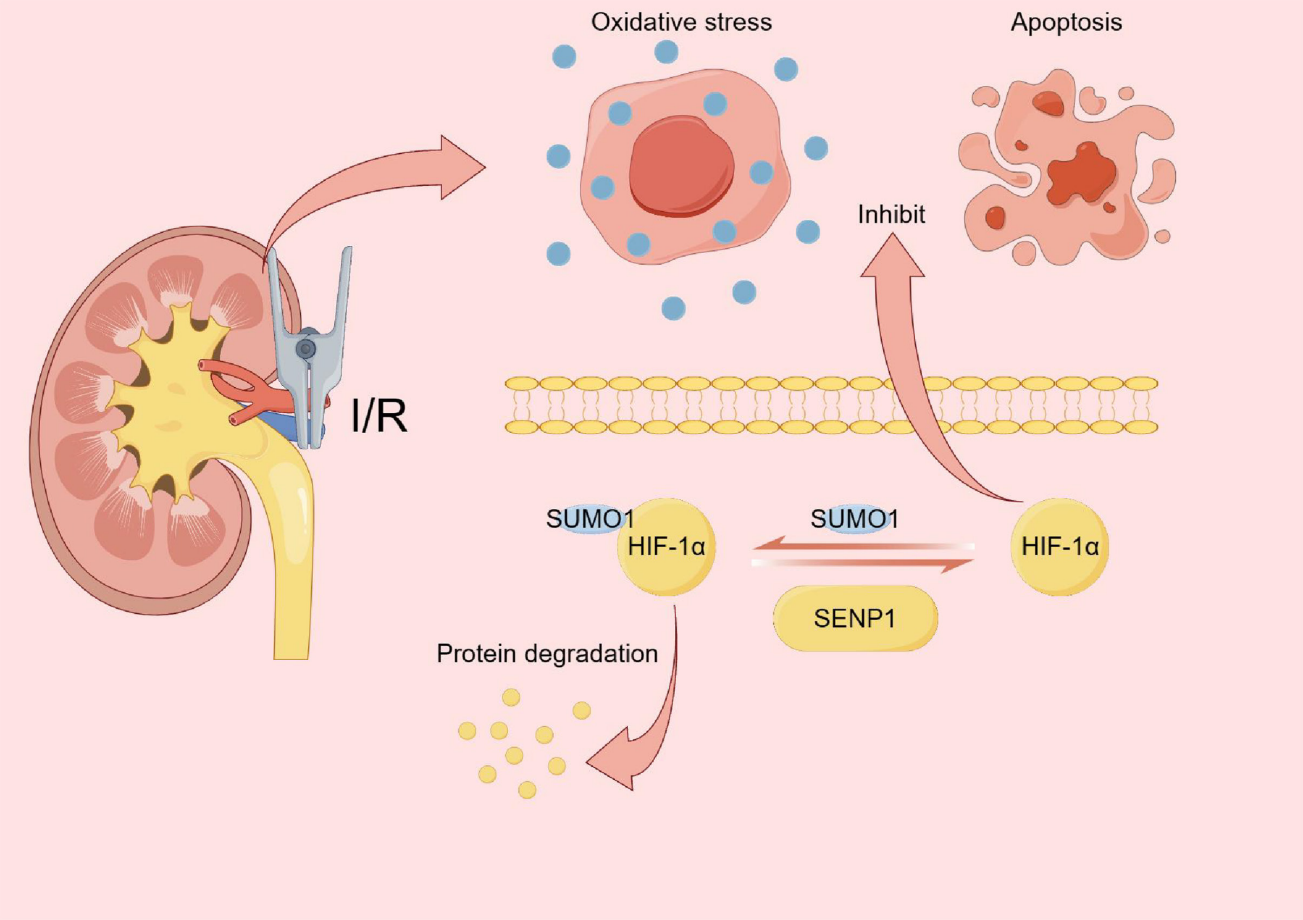
SENP1 reduced the degradation of HIF‐1α and attenuated oxidative stress and apoptosis in RIRI by regulating the deSUMOylation of HIF‐1α.

In this study, we observed that SENP1 enhanced the stability of HIF‐1α by deSUMOylation in hypoxic conditions. Moreover, the overexpression of SENP1 effectively attenuated oxidative stress and apoptotic pathways in the context of RIRI. Importantly, our study established HIF‐1α as a direct target protein of SENP1, highlighting the direct involvement of HIF‐1α in mediating the protective effects conferred by SENP1 (Figure 8). Consequently, our findings suggested that SENP1 could serve as a potential therapeutic target for RIRI, introducing novel avenues for its treatment.

RIRI stood as a primary instigator of acute kidney injury (AKI), commonly stemming from surgical procedures and transplantations.^1^ This injury typically manifested as tubular damage. Existing studies had linked SENP1 to various conditions such as tumours, lung injury and brain injury.^10^ The decrease in SENP1 expression elevates the apoptosis rate in human glioblastoma cells,^25^ while its deficiency inhibits the NF‐κB signalling pathway, resulting in apoptosis of tumour cells in multiple myeloma.^26^ However, investigations regarding SENP1's involvement in RIRI are currently lacking.

In our investigation, we initially established a RIRI model to observe a notable increase in SENP1 expression during this injury. Subsequently, we demonstrated that the overexpression of SENP1 played a mitigating role in RIRI. A substantial body of evidence underscored the significance of oxidative stress and apoptosis as pivotal mechanisms in RIRI. Apoptosis, constituting a form of programmed cell death, had been identified as a hallmark process in the context of RIRI.^27^ When cells encountered detrimental stimuli, a surge in ROS production occurred, disrupting the delicate balance between oxidation and antioxidant defences, ultimately culminating in oxidative stress.^3^ This accumulation of ROS stemmed from aberrant signalling pathways, inflammatory infiltrated, cellular dysfunction and renal cell death, collectively contributing to acute kidney injury (AKI).^28^ In our study, we observed an upregulation of apoptotic proteins such as BAX and C‐Caspase3 following RIRI. Simultaneously, antioxidant enzyme proteins like SOD1, SOD2 and CATLASE exhibited a decrease post‐injury and hypoxia. Notably, the overexpression of SENP1 reversed this process, while conversely, the knockdown of SENP1 exacerbated these effects. Consequently, our findings suggested that the renoprotective impact of SENP1 overexpression may manifest through the inhibition of oxidative stress and apoptosis.

HIF, comprising HIF‐1, 2 and 3, assumes a pivotal role in cellular adaptation to hypoxia, with predominant expression of HIF‐1α in renal tubular epithelial cells and of HIF‐2α in interstitial, endothelial and fibroblast cells.^29^ HIF‐1α orchestrates pivotal processes including angiogenesis, erythropoiesis, cell proliferation, survival and apoptosis, facilitating cellular survival under hypoxic conditions.^30^ The partial protective effects of HIF‐1α in RIRI are mediated through increased expression of its target genes, involved in metabolic shifts from glucose metabolism to glycolysis, ROS scavenging and modulation of cell survival. Nonetheless, the mechanisms underlying HIF‐1α's actions in RIRI are multifaceted.^31^, ^32^, ^33^ Ubiquitination‐mediated modulation of HIF‐1α has been scrutinized in RIRI, influencing its pathophysiological processes.^34^ But increasing evidence suggested the significance of HIF‐1α as a crucial SUMO substrate, pivotal in responding to hypoxia and subject to regulation through processes such as deSUMOylation.^9^, ^35^, ^36^, ^37^ SUMOylated HIF‐1α exhibited diverse roles, and members of the SENP family, including SENP1, participated in deSUMOylation binding proteins. In breast cancer, suppression of SENP1 activity diminishes deSUMOylation of HIF‐1α, culminating in HIF‐1α degradation and inhibition of cancer metastasis.^38^ Under hypoxic conditions, upregulation of SENP1 expression in podocytes fosters the stabilization and activation of HIF‐1α, promoting the survival of glomerular endothelial cells (GEnC) and angiogenesis as a countermeasure against hypoxia.^39^ Our study demonstrated that SENP1, specifically in HK2 cells, deSUMOylated HIF‐1α, thereby enhancing the stability of HIF‐1α. Knockdown of SENP1 resulted in an increase and accumulation of SUMOylation of HIF‐1α under hypoxic or ischemic conditions. Conversely, overexpression of SENP1 led to a decrease in SUMOylated HIF‐1α. Further investigations revealed that the protective effect of SENP1 overexpression against RIRI was compromised in the presence of a HIF‐1α inhibitor. This suggested that HIF‐1α functions as a target molecule for SENP1, mediating the role of SENP1 in RIRI. Currently, HIF‐1 prolyl hydroxylase (PH) inhibitors, as a new generation of approved oral therapeutic agents for renal anaemia, correct anaemia by activating the HIF pathway and targeting the HIF pathway has become a therapeutic strategy for a wide range of diseases.^40^, ^41^ Our study demonstrated that SENP1 has the ability to stabilize HIF‐1α, which provides new ideas and insights for the development of SENP1‐related drugs.

In conclusion, our study unveiled a novel role for SENP1 in mitigating RIRI. We observed that the overexpression of SENP1 effectively mitigated oxidative stress and apoptosis during RIRI through the deSUMOylation of HIF‐1α. These findings highlighted SENP1 as a potential therapeutic target for the treatment of RIRI, offering new insights into potential avenues for intervention.

## AUTHOR CONTRIBUTIONS

**Yumin Hui:** Conceptualization (lead); data curation (lead); formal analysis (equal); validation (equal); visualization (equal); writing – original draft (lead). **Kang Xia:** Conceptualization (equal); data curation (equal); methodology (equal); resources (equal); software (equal); writing – original draft (equal). **Jiacheng Zhong:** Investigation (equal); methodology (equal); project administration (equal); resources (equal); software (equal). **Ye Zhang:** Project administration (equal); resources (equal); writing – original draft (equal). **Qiangmin Qiu:** Data curation (equal); methodology (equal); resources (equal); software (equal); validation (equal). **Zhiyuan Chen:** Funding acquisition (equal); methodology (equal); project administration (equal); supervision (equal). **Lei Wang:** Formal analysis (equal); funding acquisition (equal); methodology (equal); supervision (equal); writing – original draft (equal). **Xiuheng Liu:** Funding acquisition (equal); methodology (equal); project administration (equal); resources (equal); supervision (equal); writing – original draft (equal).

## CONFLICT OF INTEREST STATEMENT

The authors declare that they have no known competing financial interests or personal relationships that could have appeared to influence the work reported in this paper.

## Supporting information


**Table S1.** Sequences of primers used for quantitative real‐time PCR analysis.

## Data Availability

The data that support the findings of this study are available from the corresponding author upon reasonable request.
