# Supplementary material for: SENP1 reduces oxidative stress and apoptosis in renal ischaemia–reperfusion injury by deSUMOylation of HIF‐1α
Source: J Cell Mol Med. 2024 Aug 28;28(16):e70043. doi: 10.1111/jcmm.70043 (PMC11358391; doi:10.1111/jcmm.70043)
Supplement: Supplementary file 1 — Table S1. Sequences of primers used for quantitative real‐time PCR analysis. [file JCMM-28-e70043-s001.docx]

**Supplementary Table**

Table S1 Sequences of primers used for quantitative real-time PCR analysis

| Species | Gene | Forward primer (5’-3’) | Reverse primer (5’-3’) | Product length |
| --- | --- | --- | --- | --- |
| Mus musculus | Senp1 | 5‘- CTGGGGAGGTGACCTTAGTGA -3’ | 5‘- GTGATAATCTGGACGATAGGCTG -3’ | 182 bp |
|  | Bax | 5‘- CCGGCGAATTGGAGATGAACT -3’ | 5‘- CCAGCCCATGATGGTTCTGAT-3’ | 137 bp |
|  | Bcl2 | 5‘- GCTACCGTCGTGACTTCGC-3’ | 5‘- CCCCACCGAACTCAAAGAAGG-3’ | 147 bp |
|  | Casp3 | 5‘-CTCGCTCTGGTACGGATGTG-3’ | 5‘-TCCCATAAATGACCCCTTCATCA-3’ | 201 bp |
|  | Cat | 5‘-CCCCTATTGCCGTTCGATTCT-3’ | 5‘-TTCAGGTGAGTCTGTGGGTTT-3’ | 211 bp |
|  | Sod1 | 5‘-ATGGCGATGAAAGCGGTGT-3’ | 5‘-CCTTGTGTATTGTCCCCATACTG-3’ | 170 bp |
|  | Sod2 | 5‘-CAGACCTGCCTTACGACTATGG-3’ | 5‘-CTCGGTGGCGTTGAGATTGTT-3’ | 113 bp |
| Homo sapiens | BAX | 5‘- CCCGAGAGGTCTTTTTCCGAG -3’ | 5‘- CCAGCCCATGATGGTTCTGAT -3’ | 155 bp |
|  | BCL2 | 5‘- GGTGGGGTCATGTGTGTGG-3’ | 5‘- CGGTTCAGGTACTCAGTCATCC-3’ | 89 bp |
|  | CAT | 5‘-TGGAGCTGGTAACCCAGTAGG-3’ | 5‘-CCTTTGCCTTGGAGTATTTGGTA-3’ | 209 bp |
|  | SOD1 | 5‘-GGTGGGCCAAAGGATGAAGAG-3’ | 5‘-CCACAAGCCAAACGACTTCC-3’ | 227 bp |
|  | SOD2 | 5‘-GCTCCGGTTTTGGGGTATCTG-3’ | 5‘-GCGTTGATGTGAGGTTCCAG-3’ | 92 bp |
